# Manganese oxide electrode with excellent electrochemical performance for sodium ion batteries by pre-intercalation of K and Na ions

**DOI:** 10.1038/s41598-017-02028-0

**Published:** 2017-05-22

**Authors:** Mengya Feng, Qinghua Du, Li Su, Guowei Zhang, Guiling Wang, Zhipeng Ma, Weimin Gao, Xiujuan Qin, Guangjie Shao

**Affiliations:** 1 State key Laboratory of Metastable Materials Science and Technology, Yanshan University, Qinhuangdao, 066004 China; 2 Hebei Key Laboratory of Applied Chemistry, College of Environmental and Chemical Engineering, Yanshan University, Qinhuangdao, 066004 China

## Abstract

Materials with a layered structure have attracted tremendous attention because of their unique properties. The ultrathin nanosheet structure can result in extremely rapid intercalation/de-intercalation of Na ions in the charge–discharge progress. Herein, we report a manganese oxide with pre-intercalated K and Na ions and having flower-like ultrathin layered structure, which was synthesized by a facile but efficient hydrothermal method under mild condition. The pre-intercalation of Na and K ions facilitates the access of electrolyte ions and shortens the ion diffusion pathways. The layered manganese oxide shows ultrahigh specific capacity when it is used as cathode material for sodium-ion batteries. It also exhibits excellent stability and reversibility. It was found that the amount of intercalated Na ions is approximately 71% of the total charge. The prominent electrochemical performance of the manganese oxide demonstrates the importance of design and synthesis of pre-intercalated ultrathin layered materials.

## Introduction

With the excellent development of renewable and sustainable energies such as wind, tide and solar power, the smooth integration of these energies into the grid urgently needs high efficient, long cycle life, safety, low cost, large-scale energy storage systems to maximize the grid reliability, power quality and network utilization^1–6^. Among the available energy storage technologies^7–10^, battery is the best choice to store electricity in the form of chemical energy when considering the flexibility, convenience and energy conversion efficiency of device^11–13^. Lithium ion batteries have been studied most commonly as rechargeable batteries^14–18^ and magnesium ion batteries^19, 20^, zinc ion batteries^21, 22^ and aluminium ion batteries^23^ have also been investigated. Sodium-ion batteries have attracted wide attention as a promising alternative for large-scale energy storage systems, because sodium is abundant and inexpensive and has available redox potential (E (Na^+^/Na) = −2.71 V vs. SHE)^24^. Viable electrode materials are essential for the development of Na-ion batteries.

At present, there are various materials reported as cathode for sodium-ion batteries, including transition metal oxides^25, 26^, sulfides, fluorides and organic compounds^27, 28^. Transition metal oxides have been extensively investigated these years. For example, Xueqian Zhang *et al*. synthesized a layered structure Na-birnessite Na_0.58_MnO_2_·0.48H_2_O, offering a specific capacity of 80 mA h g^−1^ at 1 C^29^. Na_4_Mn_9_O_18_ synthesized by a simple solid-state route was demonstrated as a cathode material for an aqueous electrolyte sodium-ion energy storage device, having a specific capacity of 45 mA h g^−1^ ^30^. NaMnO_2_ electrode with a specific capacity of 55 mA h g^−1^ at 1 C in 2 M CH_3_COONa aqueous electrolyte for sodium-ion batteries was reported by Tarasconetal^31^. However, these researches on cathode materials cannot fully satisfy the requirements for the application of Na-ion batteries and the realization of materials with high performance is still challenging. The layered structures of A_x_MO_2+y_ (A is alkali, M is transition metal) have been extensively investigated during the 1970s and 1980s, showing unique properties compared to the non-layered structure of the compound^32^. The structures of manganese oxides can be divided into two types, the tunnel and the layer structures. The layered manganese oxides consist of stacks of sheets sharing MnO_6_ octahedra, where alkali ions are located^32, 33^. Because of the alkali ions between layers and the comparatively open structure, layered manganese oxides provide more favorable pathways for electrolyte cation diffusion than tunnel manganese oxides^34, 35^. Liqiang Mai *et al*. obtained electrochemically active layered Na_x_MnO_2_ by the use of a one-step electrodeposition technique based on the atomic-level control of material composition. The electrode presented good ionic diffusion behaviors but inferior electrochemical performance^36^. Yunlong Zhao *et al*. reported five typical cathode materials and described the synergistic effect between crystal structure and intercalated alkali metal ion, which enhanced the electrochemical performance of the compounds^37^.

In this work, we developed a facile, mild but efficient hydrothermal method to prepare ion (K^+^ and Na^+^) pre-intercalated ultrathin manganese oxide materials. The effects of pre-intercalated K and Na ions on the electrochemical performance of the manganese oxide are discussed. It is found that the material with an appropriate level of pre-intercalated ions has ultrahigh capacity, excellent cycling stability and rate performance, which attribute to its stable structure and short ion diffusion pathway. It possesses a great sodium diffusion coefficient when pre-intercalating appropriate Na ions. Two mechanisms of charge storage in the manganese oxide materials are discussed.

## Experimental

### Materials synthesis

All the reagents were bought from Tianjin Romeo chemical reagent factory. The ion pre-intercalated ultrathin manganese oxide materials were synthesized by a one-step hydrothermal method. Firstly, KMnO_4_ (0.1054 g) was dissolved in deionized water (30 mL), then Na_2_SO_4_ was dissolved in the above solution (The concentration ratios of Na_2_SO_4_ and KMnO_4_ were 0:1, 7.5:1, 15:1 and 22.5:1). Subsequently, 20 mL of MnSO_4_ (0.1690 g) aqueous solution was dropped into the mixture. After stirred vigorously, the resultant dark brown solution was poured into a 60 mL Teflonlined autoclave and then kept at 160 °C for 1 h in an oven. After the autoclave cooled to room temperature naturally, the product was filtered out, washed with deionized water and ethanol for several times and dried at 80 °C for 12 h. The products prepared by 0:1, 7.5:1, 15:1 and 22.5:1 concentration ratio of Na_2_SO_4_ and KMnO_4_ were labeled as M0, M1, M2 and M3, respectively.

### Structural and physical characterization

XRD measurement (D-max-2500/PC X-ray Diffractometer) was performed to study the crystalline structure of the products. ICP was carried out to analyze the element content of the compounds. The morphology and structural properties of the materials were studied by scanning electron microscopy (SEM) (S-4800) and transmission electron microscopy (TEM) (JEM-2010). Fourier-transformed infrared (FTIR) absorption spectra were taken using a 60-SXB IR spectrometer. Raman spectra were recorded by a Renishaw RM-1000 laser Raman microscopy system.

### Electrochemical characterization

Working electrodes were prepared by mixing the manganese oxide powder, as active material, with polytetrafluoroethylene (PTFE) and acetylene black in a mass ratio of 80:10:10. PTFE was first added to ethanol. Then 16 mg manganese oxide powder and 2 mg acetylene black were added into the mixture and dispersed by ultrasonic stirring for 15 min. The prepared slurry was then coated onto a stainless steel mesh. The electrode was dried at 80 °C in air for 12 h. The electrode was then pressed using a pelletizer under 10 ton. A three electrode system was assembled by using the stainless steel mesh coated with manganese oxide as the working electrode, a Pt-foil as the counter and a saturated calomel electrode (SCE) as the reference. And cyclic voltammetry (CV) and electrochemical impedance spectra (EIS) were applied to characterize the electrochemical properties of the electrode, which were performed on a CHI 660E electrochemical workstation (Chenhua, Shanghai China). Galvanostatic cycling performance of the electrode was evaluated by using a potentiostat/galvanostat (NEWARE, Shenzhen China). CV was done between 0 and 1 V in 1 M Na_2_SO_4_ electrolyte at different scan rates. The charge/discharge cycling measurements were recorded at the same potential range. Cells were cycled using a three electrode system at room temperature at rates of 1.5 C to 50 C (1 C = 200 mAg^−1^ applied current density). The electrochemical impedance spectra (EIS) were carried out over a frequency range of 0.01 Hz to 100 000 Hz with an AC modulation of 5 mV. All the aforementioned tests were performed at room temperature.

## Results and Discussion

**Figure 1:**
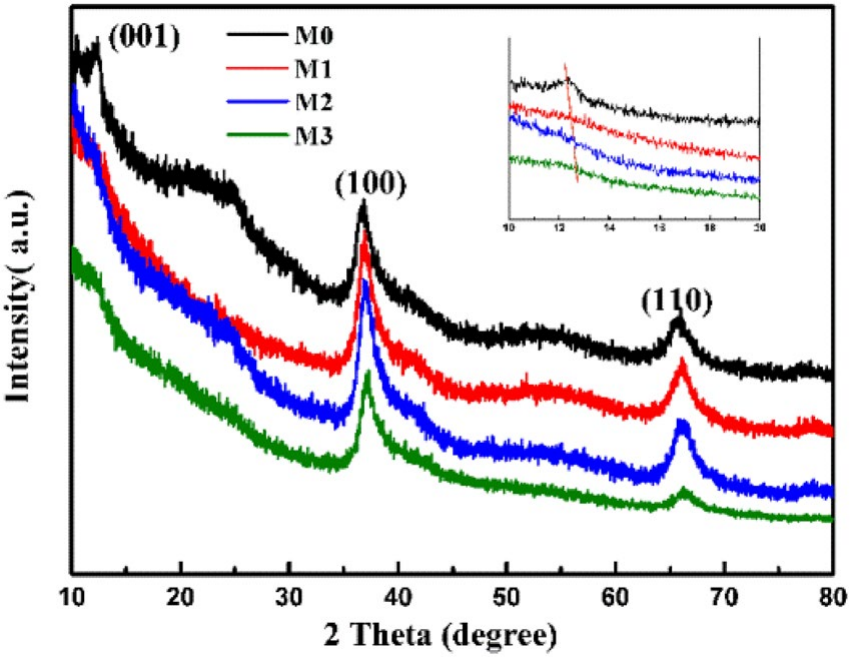
XRD patterns of the as-prepared manganese oxides.

Figure 1 shows the XRD patterns of all the as-prepared samples. According to the XRD patterns, the peaks at about *2θ* = 12°, 37° and 66° corresponding to (001), (100) and (110) crystal planes are indexed to a δ-type manganese oxide^38^. The obvious features in the figure are the broad peaks, indicating a poorly crystalline phase. The peaks corresponding to (001), (100) and (110) crystal planes of M1, M2 and M3 shift to a higher diffraction angle, which indicates that the interplanar spacing of the materials become smaller. These results are shown further in Table S1. It shows ICP results of the molar ratio for Na and K in the samples. I can be see that K ions are replaced by Na ions with the increase in the ratio of Na_2_SO_4_ and KMnO_4_ for M1, M2 and M3 and the interplanar spacing of the materials become smaller and smaller. As a portion of K ions are replaced by Na ions that have a smaller radius than K ions, so the interplanar spacing becomes smaller.

**Figure 2:**
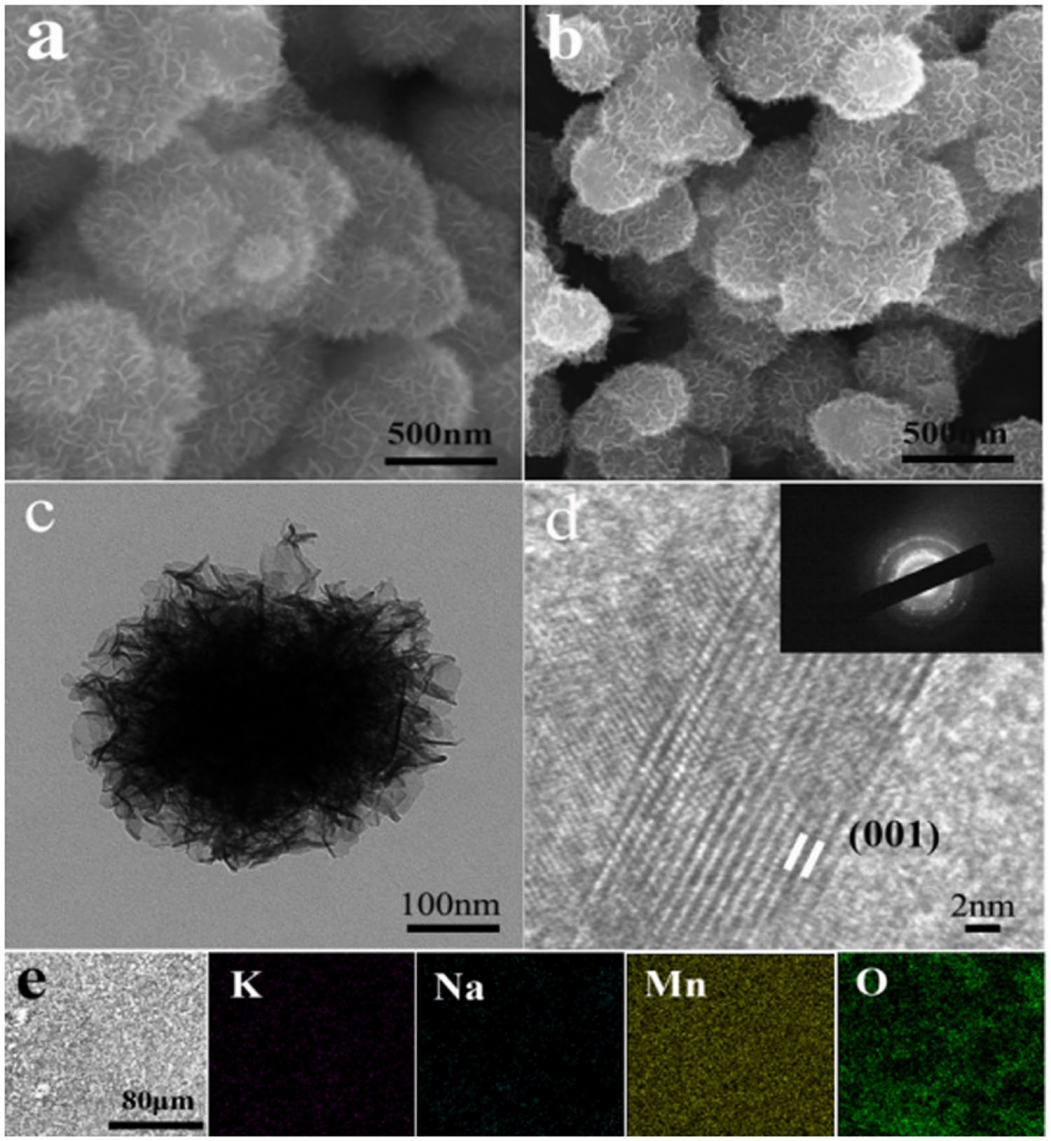
SEM images of (**a**) M0 and (**b**) M2, (**c**) TEM image of M2, (**d**) HR-TEM and SAED images of M2 and (**e**) EDS elemental mapping of M2.

SEM and TEM were performed to characterize the morphology of the as-prepared samples. All the samples show a flower-like structure, built from plenty of nanosheets (Fig. S1). Figure 2a,b present the SEM images of M0 and M2, respectively. As we can see, the flower-like structures with connected nanosheets of M0 and M2 are about ca.500 nm and ca.400 nm in diameter, respectively. To prove the detailed morphology and structure, the TEM of M2 was analyzed. As shown in Fig. 2c, many nanosheets stretch out towards the edges of the flower-like structure. The HR-TEM image (Fig. 2d) clearly demonstrates that the interplanar spacing is about 0.70 nm. The polycrystallinity is also confirmed by SAED pattern (inset of Fig. 2d). The ultrathin flower-like structure of the nanosheets is critical to increase the material-electrolyte contact area and enhance ion diffusion in the process of charging and discharging. Meanwhile, the distribution of constitutive elements of M2 was analyzed by EDS elemental mapping as shown in Fig. 2e. It clearly supports a homogeneous dispersion of pre-intercalated K ions and Na ions in the synthesized material.

**Figure 3:**
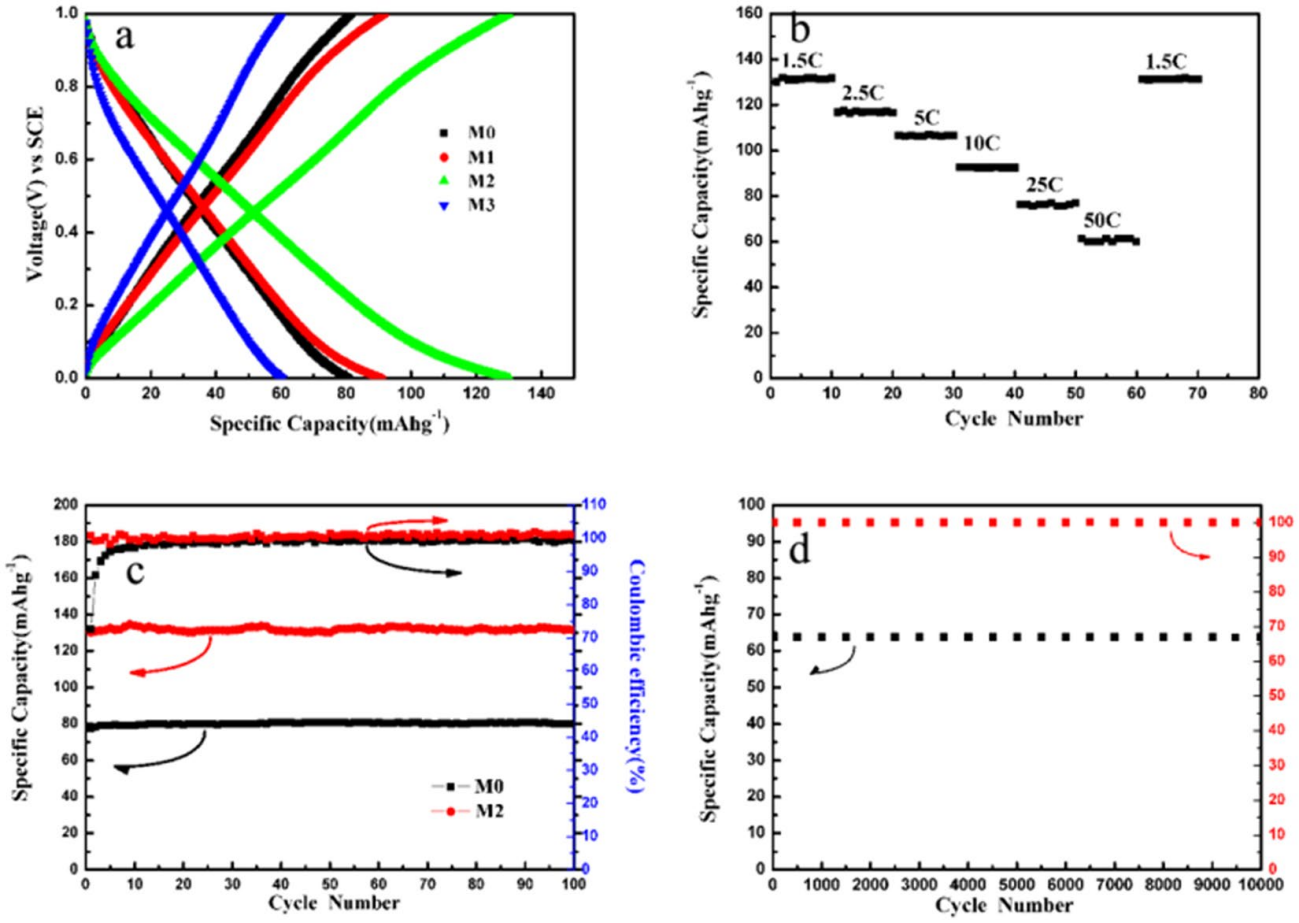
(**a**) Charge-discharge profiles of the as-prepared manganese oxides at 1.5 C, (**b**) Rate performance of M2 electrode, (**c**) Cycling performance of M0 and M2 electrodes at 1.5 C, and (**d**) Long cycling stability of M2 electrode at 50 C.

In order to reveal the pre-intercalation influence on the electrochemical performance of the as-prepared manganese oxides, galvanostatic charge–discharge measurements were performed in the above mentioned three-electrode system. Figure 3a shows the charge-discharge profiles of the tenth cycle of all the as-prepared samples at 1.5 C in 1 M Na_2_SO_4_ electrolyte. It shows discharge capacities of 79.7, 89.7, 133.8 and 58.9 mA h g^−1^ for M0, M1, M2 and M3, respectively. The voltage plateaus on the charge–discharge curves are not obvious, which is probably due to the ultrathin nanosheet structure capable for extremely rapid intercalation and de-intercalation of Na ions during charging and discharging. Besides, the pre-intercalation of Na ions facilitates the efficient access of electrolyte ions and shortens the ion diffusion pathways. The capacity of M3 is lower than that of M2, which may be due to smaller interplanar spacing that impedes the diffusion of Na ions in the electrolyte. Figure 3b shows the rate capability of M2. The specific discharge capacities are about 133.8, 116.5, 106.6, 92.3, 76.3 and 61.4 mA h g^−1^ at 1.5 C, 2.5 C, 5 C, 10 C, 25 C and 50 C, respectively. The capacity can be recovered to the original value when the current density is turned back from 50 to 1.5 C. These results indicate the excellent rate performance of the materials. Figure 3c presents cycling performance of M0 and M2. The coulombic efficiency decayed by only 1% after 100 cycles for M0, and it is retained at nearly 100% for M2. In addition, M2 exhibits prominent cycling performance in 10000 cycles and excellent stability with almost 100% capacity retention after 10000 cycles, as shown in Fig. 3d. The excellent cycling performance is due to the pre-intercalations of K and Na ions. The former is provided to support and stabilize the layered structure, while the latter facilitates the efficient access of electrolyte ions and shortens the ion diffusion pathways. Table 1 compares our results with some manganese oxides reported recently for aqueous electrolyte batteries. It can be seem that the reversible capacity of M2 synthesized in the present work is greatly higher than the reported Na_0.58_MnO_2_·0.48H_2_O^29^, Na_4_Mn_9_O_18_^30^, NaMnO_2_^31^, K_0.27_MnO_2_^38^, Na_0.95_MnO_2_^39^ and Na_0.44_MnO_2_^40^.

**Table 1:** The electrochemical performance of various electrodes in aqueous electrolytes.

Active material	Capacity (mA h g^−1^)	Current density	Efficiency (%)	Ref.
M2	133.8	1.5 C	100	This work
Na_0.58_MnO_2_·0.48H_2_O	80.0	1 C	60	29
Na_4_Mn_9_O_18_	45	0.125 C	—	30
NaMnO_2_	60	1 C	91.5	31
K_0.27_MnO_2_	64.7	0.2 A g^−1^	74	38
Na_0.95_MnO_2_	60.0	2 C	92	39
Na_0.44_MnO_2_	40	0.1 C	—	40

**Figure 4:**
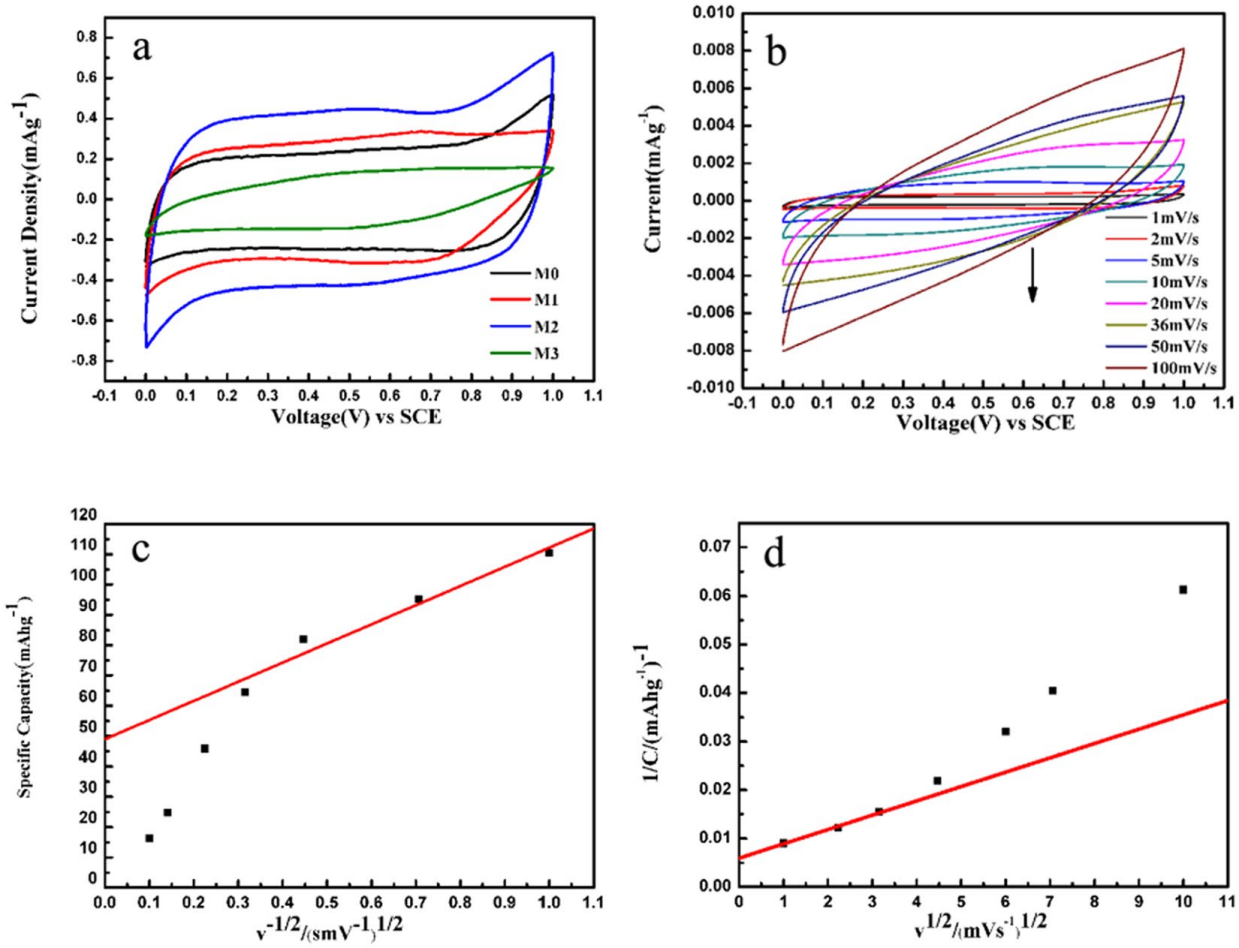
(**a**) CV curves of the as-prepared manganese oxide electrodes at scan rate of 1 mV s^−1^, (**b**) CV curves of M2 electrode at different scan rates, (**c**) The relationship of specific capacitance versus inverse square root of scan rate, and (**d**) The relationship of inverse specific capacity versus square root of scan rate.

The electrochemical performance of the manganese oxide electrodes was further evaluatedby cyclic voltammetry. Figure 4a shows the CV curves of all the as-prepared manganese oxide electrodes at the potential ranging from 0 to 1 V at scan rate of 1 mV s^−1^ in 1 M Na_2_SO_4_ electrolyte. As we can see from the area of the CV plot, the specific capacity of M2 is much higher than that of the other samples^41^. Figure 4b shows the CV curves of M2 electrode at scan rates from 1 to 100 mV s^−1^ in 1 M Na_2_SO_4_ electrolyte. The redox peaks between 0 to 1 V (vs. SCE) are not obviously observed, which suggests the extremely rapid intercalation/de-intercalation of Na ions. It may be due to the ultrathin layered structure with appropriate pre-intercalation of Na ions. To investigate electrochemical characteristics of the manganese oxide electrodes, we used a method developed by Trasatti to separate capacitive elements from intercalation processes here^42, 43^. Two charge storage mechanisms were proposed for manganese oxide electrodes. The first one is the intercalation of alkali metal cations (C^+^), such as Na^+^, during reduction and de-intercalation on oxidation in the electrode^44^:

1 $${{\rm{MnO}}}_{2}+{{\rm{C}}}^{+}+{{\rm{e}}}^{-}\leftrightarrow {{\rm{MnOOC}}}^{+}$$

The second one is that the cations in the electrolyte are adsorbed at the manganese oxide electrode^45^:

2 $${({{\rm{MnO}}}_{2})}_{{\rm{surface}}}+{{\rm{C}}}^{+}+{{\rm{e}}}^{-}\leftrightarrow {({{\rm{MnO}}}_{2}-{{\rm{C}}}^{+})}_{{\rm{surface}}}$$

We can quantitatively distinguish the capacitive contribution to the current response through a closer examination of cyclic voltammetry. According to the concepts described above, we can express the current response at a fixed potential as being the combination of two separate mechanisms, namely the outer charge and diffusion-controlled Na^+^ insertion^42, 46^:

3 $$i(V)={k}_{1}+{k}_{2}{v}^{1/2}$$

For analytical purposes, we rearrange the equation to

4 $$i(V)/{v}^{1/2}={k}_{1}/{v}^{1/2}+{k}_{2}$$

In Eq. 3, *k*_1_ *v* and *k*_2_ *v* ^1/2^ correspond to the current contributions from outer charge and the diffusion controlled Na^+^ insertion processes, respectively. The values of *k*_*1*_ and *k*_*2*_ were obtained from the slope and intercept of the straight line, respectively. This method was used to distinguish the currents arising from Na^+^ insertion and those from capacitive effects. The actual contribution of the adsorption can be figured out, based on the CV, by extrapolating the voltammetric charge at scan rate to infinity and calculating the linear dependence of the voltammetric charge on the inverse square root of scan rated. At high scan rates, the access of the protons to the inner surface of the electrode material becomes slow due to the limiting diffusion. Then the electrochemical response depends only on the outer active surface^44^. The outer charge is given in the extrapolation plot in Fig. 4c. The capacity related to the outer and more accessible active surface is calculated to be 48.9 mA h g^−1^. At low scan rates, there is enough time for protons diffusing to the inner surface. The total charge is given in the extrapolation plot in Fig. 4d. The capacity due to the whole active surface is calculated to be 168.3 mA h g^−1^ including the outer and inner active surfaces. The value of the outer charge in the manganese oxide electrodes is approximately 29% of the total charge. Namely, the value of Na ion intercalation is approximately 71% of the total charge.

**Figure 5:**
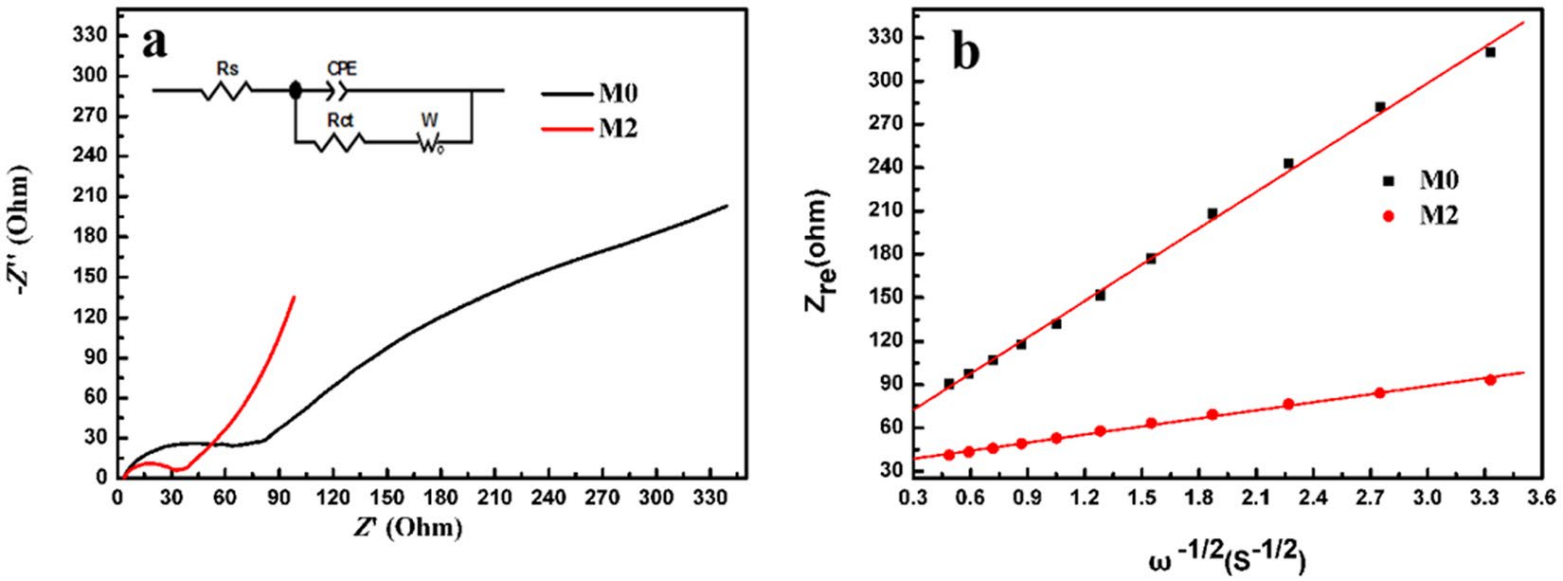
(**a**) Nyquist plots of M0 and M2 electrodes. The inset image is the corresponding equivalent circuit. (**b**) Relationship between Z_re_ and ω^−1/2^ of M0 and M2 electrodes at low frequencies.

Figure 5a displays the Nyquist plots of the M0 and M2 electrodes in 1 M Na_2_SO_4_ electrolyte. As we can see, both the impedance curves contain a semicircle and a line. The start of the semicircle intersects with the real impedance axis, representing the electrolyte resistance. The semicircle is ascribed to the charge transfer process and the line at lower frequency region is attributed to the ion diffusion in the manganese oxide electrode. In the low frequency region, straight lines are obtained. The steeper the straight line is, the more rapid the ion diffusion of the electrode materials is. The Nyquist plots are fitted to an equivalent circuit (the inset of Fig. 5a) and the derived impedance parameters are listed in Table 2. Both the charge transfer resistance, R_ct_, and the diffusion resistance, Z_w_, of M2 are obviously lower than those of M0. It demonstrates that the pre-intercalation of Na ions can greatly increase the electrochemical performance of M2. In low frequencies, the straight line region represents Warburg diffusion of Na ions in the manganese oxide electrode. Warburg diffusion coefficient can be calculated from the following formula:

5 $${{\rm{Z}}}_{{\rm{re}}}={{\rm{R}}}_{{\rm{e}}}+{{\rm{R}}}_{{\rm{ct}}}+{{\rm{\sigma }}{\rm{\omega }}}^{-1/2}$$

where R_e_ is the electrolyte resistance, R_ct_ is the transfer resistance and ω is the angular frequency. So Warburg diffusion coefficient, σ, is the slope of the line. The Na ion diffusion coefficient, D, can be calculated from the following formula further^47^:

6 $${\rm{D}}={{\rm{R}}}^{2}{{\rm{T}}}^{2}/2{{\rm{A}}}^{2}{{\rm{n}}}^{4}{{\rm{F}}}^{4}{{\rm{C}}}^{2}{{\rm{\sigma }}}^{2}$$

where R is the gas constant, T is the room temperature, A is the surface area of the electrode, n is the number of the electrons per molecule in the reaction, F is the Faraday constant, and C is the concentration of Na ions in the electrode. Σ was calculated from the line of Zre ~ ω^−1/2^ (shown in Fig. 5b). The Na diffusion coefficients of M0 and M2 were calculated to be 1.0665 × 10^−15^ cm^2^ s^−1^ and 5.2476 × 10^−14^ cm^2^ s^−1^, respectively. It is obvious that M2 has a greater Na ion diffusion coefficient. It is attributed to the appropriate pre-intercalation of K ions and Na ions.

**Table 2:** The derived impedance parameters of M0 and M2.

Active material	R_s_(Ω)	R_ct_(Ω)	Z_w_(Ω)
M0	3.8	18.2	0.079
M2	2.0	12.1	0.018

**Figure 6:**
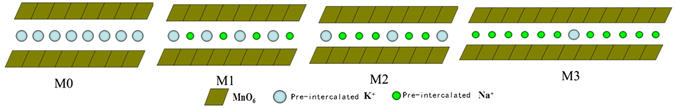
Schematic diagram of manganese oxides with different ratios of pre-intercalated Na ions.

According to the above analysis, the as-prepared manganese oxides are presented by schematic diagrams in Fig. 6. The interplanar spacing of the materials becomes more and more small with the increase in the ratio of Na_2_SO_4_ and KMnO_4_. For M0, there is no pre-intercalated Na ions, but K ions residing in the layer support and stabilize the ultrathin structure. For M1, some K ions are replaced by Na ions, leading to a smaller interplanar spacing. For M2, more K ions are replaced by Na ions, and the appropriate pre-intercalation of Na ions facilitates the efficient access of electrolyte ions, shortens the Na ions diffusion pathways and, as a result, improves the electrochemical performance. However, when overmuch K ions are replaced by Na ions, for example, in M3, the interplanar spacing becomes so small that it is difficult for Na ions to diffuse, leading to a poor specific capacity.

## Conclusions

In summary, we used a facile but efficient hydrothermal method to prepare K and Na ion pre-intercalated manganese oxides with a flower-like ultrathin layered structure, which exhibits excellent electrochemical performance in the three-electrode system using 1 M Na_2_SO_4_ aqueous solution as the electrolyte. The capacity (133.8 mA h g^−1^) of K and Na ion pre-intercalated manganese oxide is much higher than that (79.7 mA h g^−1^) of only K ion pre-intercalated manganese oxide and it presents excellent rate performance. The K and Na ion pre-intercalated manganese oxide delivers an excellent cycling capability with a capacity retention rate of almost 100% after 10000 cycles. Additionally, the discharge mechanism was studied. The value of Na ions intercalated is calculated to be approximately 71% of the total available charge. The hydrothermal method with pre-intercalation of alkali metal ions was demonstrated to be an effective technique for improving the performance of manganese oxide materials for applications in Na batteries.

## Electronic supplementary material

Supplementary Information
