# Supplementary material for: Manganese oxide electrode with excellent electrochemical performance for sodium ion batteries by pre-intercalation of K and Na ions
Source: Sci Rep. 2017 May 22;7:2219. doi: 10.1038/s41598-017-02028-0 (PMC5440409; doi:10.1038/s41598-017-02028-0)
Supplement: Supplementary file 1 — Supplementary Information [file 41598_2017_2028_MOESM1_ESM.doc]

**Manganese oxide electrode with excellent electrochemical performance for sodium ion batteries by pre-intercalation of K and Na ions**

Mengya Feng^1,2^, Qinghua Du^1,2^, Li Su^1,2^, Guowei Zhang^1,2^, Guiling Wang^1,2^, Zhipeng Ma^1,2^, Weimin Gao^2*^, Xiujuan Qin^1,2*^，Guangjie Shao^1,2*^

^1^ Hebei Key Laboratory of Applied Chemistry, College of Environmental and Chemical Engineering, Yanshan University, Qinhuangdao 066004, China;

^2^ State key Laboratory of Metastable Materials Science and Technology, Yanshan University, Qinhuangdao 066004, China.

**Supporting Information**

**
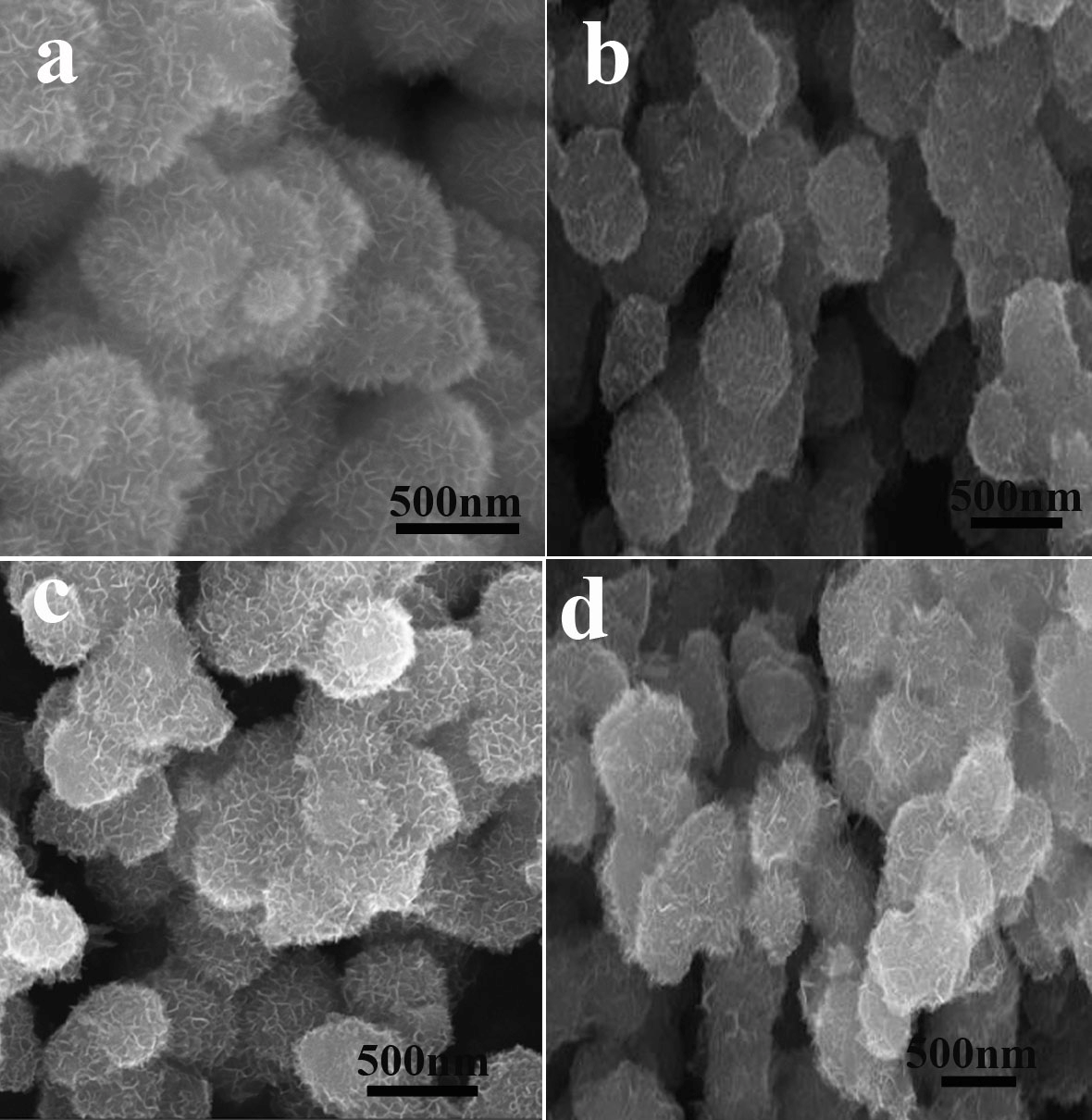
**

**Fig.S1 SEM images of the as-prepared manganese oxides.**

**Table S1. The molar ratio of the ICP results for Na and K in the as-prepared samples and interplanar spacing with the increasing ratio of Na2SO4 and KMnO4**

| **Active material** | **Na: K** | **2θ(**°**)** | **d(nm)** |
| --- | --- | --- | --- |
| **M0** | **0** | **12.39** | **0.7139** |
| **M1** | **1.2** | **12.43** | **0.7115** |
| **M2** | **2.5** | **12.56** | **0.7042** |
| **M3** | **11.0** | **12.67** | **0.6984** |

**
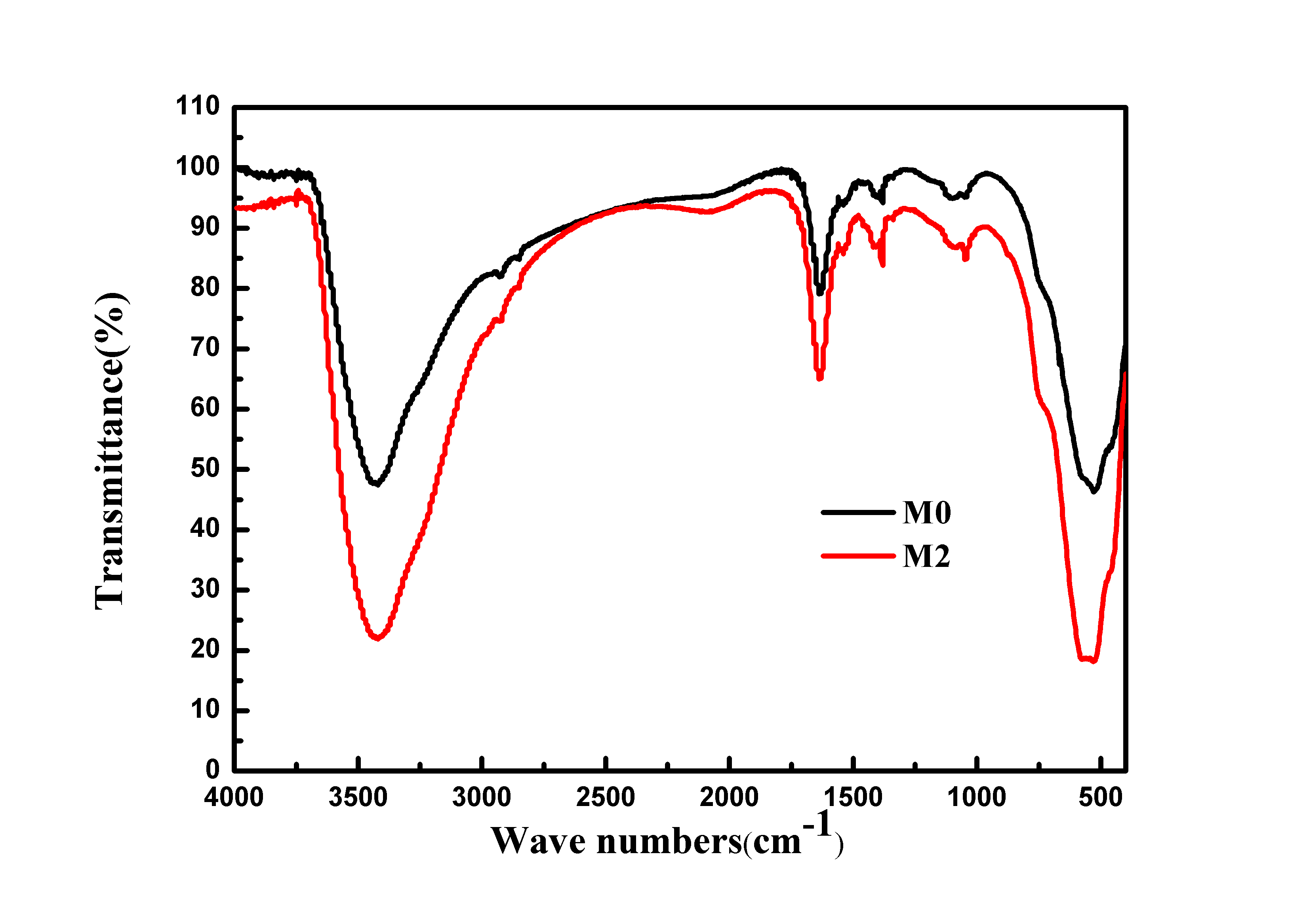
**

**Fig.S2** **FTIR spectra of the as-prepared manganese oxides.**

**
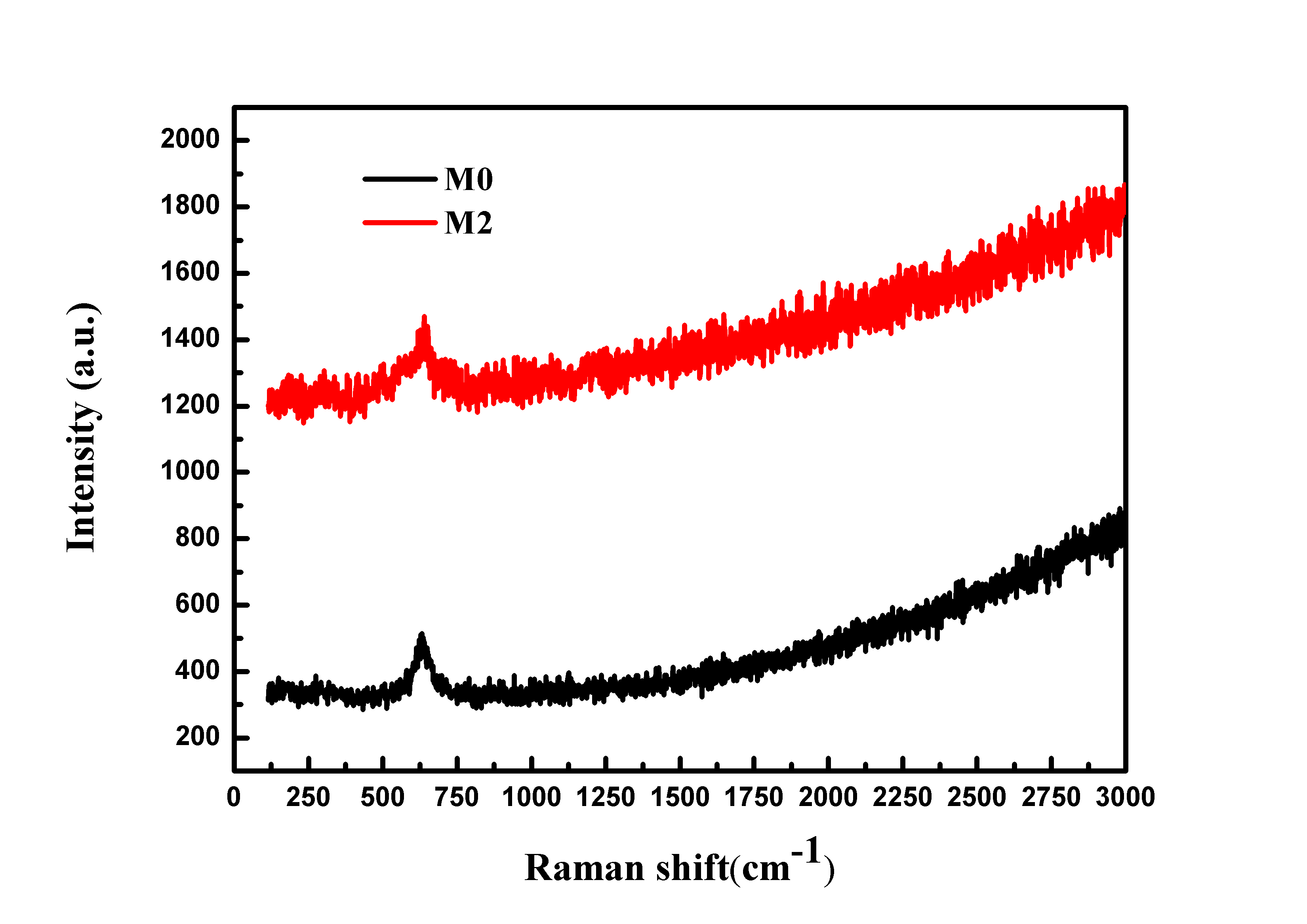
**

**Fig.S3 Raman spectra of the as-prepared manganese oxides.**

FTIR spectroscopy in Fig.S3 was carried out to discuss the change in bonding associated with M0 and M2. From the FTIR spectra, it can be investigated that a broad band arises at around 3420 cm^-1^ for both samples, which is caused by hydroxyl groups bound to the alkali metal ions and some crystal water in the layers. Furthermore, other bands at 1640, 1370, and 1100 cm^-1^ for both samples are due to O–H bending vibrations combined with Mn atoms. The absorption band at 570 cm^–1^ is assigned to stretching vibrations of the Mn-O and/or Mn–O–Mn in [MnO_6_] octahedral. The FTIR shows that pre-intercalation with K ions and Na ions has not damaged the structure of as-prepared manganese oxide, but it is believed to be conducive to enhance its electrochemical performance. The Raman spectra in Fig.S4 demonstrate a consistent conclusion. The Raman band at 638 cm^-1^ for both samples is attributed to the symmetric Mn–O stretching vibration of [MnO_6_] octahedral. It confirms the results further that the pre-intercalation with K ions and Na ions has not damaged the structure of as-prepared manganese oxide.
